# Continuous research monitoring improves the quality of research conduct and compliance among research trainees: internal evaluation of a monitoring programme

**DOI:** 10.12688/aasopenres.13117.1

**Published:** 2020-11-25

**Authors:** Mirriam Akello, Sarah Coutinho, Mary Gorrethy N-Mboowa, Victoria D Bukirwa, Agnes Natukunda, Lawrence Lubyayi, Grace Nabakooza, Stephen Cose, Alison M. Elliott

**Affiliations:** 1 Medical Research Council/Uganda Virus Research Institute and London School Hygiene Tropical Medicine Uganda Research Unit, Entebbe, Uganda; 2 Makerere University/ Uganda Virus Research Institute Centre of Excellence in Infection and Immunity Research and Training (MUII-Plus), Uganda Virus Research Institute, Entebbe, Uganda; 3 Department of Immunology and Molecular Biology, Makerere University, Kampala, Uganda; 4 Centre for Computational Biology, Uganda Christian University, Mukono, Uganda; 5 Clinical Research Department, London School of Hygiene & Tropical Medicine, London, UK

**Keywords:** Internal monitoring, Good Clinical Research Practice, trainees or investigators, Uganda, Africa, research quality

## Abstract

**Background: **Research site monitoring (RSM) is an effective way to ensure compliance with Good Clinical Practice (GCP). However, RSM is not offered to trainees (investigators) at African Institutions routinely. The Makerere University/Uganda Virus Research Institute Centre of Excellence in Infection and Immunity Research and Training (MUII-Plus) introduced internal monitoring to promote the quality of trainees’ research projects. Here, we share our monitoring model, experiences and achievements, and challenges encountered.

**Methods: **We analysed investigators’ project reports from monitoring visits undertaken from April 2017 to December 2019. Monitors followed a standard checklist to review investigator site files and record forms, and toured site facilities. We planned four monitoring visits for each trainee: one at site initiation, two interim, and a closeout monitoring visit. A team of two monitors conducted the visits.

**Results: **We monitored 25 out of the 26 research projects in progress between April 2017 and December 2019. Compliance with protocols, standard operating procedures, GCP, and GCLP improved with each monitoring visit. Median (IQR) compliance rate was 43% (31%, 44%) at site initiation visit for different monitoring items, 70% (54%, 90%) at the 1st interim monitoring visit, 100% (92%, 100%) at 2nd interim monitoring visit and all projects achieved 100% compliance at site closeout.  All investigators had good work ethics and practice, and appropriate facilities. Initially, some investigators’ files lacked essential documents, and informed consent processes needed to be improved. We realized that non-compliant investigators had not received prior training in GCP/GCLP, so we offered them this training.

**Conclusions: **Routine monitoring helps identify non-compliance early and improves the quality of research. We recommend continuous internal monitoring for all research studies. Investigators conducting research involving human subjects should receive GCP/GCLP training before commencing their projects. Institutional higher degrees and research ethics committees should enforce this as a requirement for project approvals.

## Background

Research site monitoring (RSM) is a systematic process that involves the close supervision of an investigator to ensure that all research activities are implemented according to the approved study protocols and good clinical practice (GCP).

All studies that involve humans subjects must be reviewed ethically and scientifically before their start ^1– 5^ and monitored as per international human research regulatory guidelines ^4^. Research Ethics Committees are critical in giving independent corrective review of proposed studies to ensure that the dignity and wellbeing of potential participants are fully protected ^1^. These committees review study tools such as consent and data collection forms and laboratory and data analysis protocols, to ensure that they align with GCP and Good Clinical Laboratory Practice (GCLP) guidelines ^4, 6^.

Research site initiation procedures and routine monitoring of ethically approved human studies are essential to ensure that all investigators are qualified and competent to undertake the proposed work, required study facilities and tools are available, participants’ rights and safety are protected during data collection, and data is collected accurately to produce reliable results ^4^. Additionally, continuous monitoring prevents research fraud, minimizes un-ethical practices, enables early detection of protocol deviations, and ensures rightful and effective dissemination of research results ^4, 6, 7^.

The Makerere University/Uganda Virus Research Institute Centre of Excellence in Infection and Immunity Research and Training (MUII-Plus) is a program under the African Academy of Sciences DELTAS Initiative, whose goal is to promote scientific quality and to train future research leaders for excellence ( www.muii.org.ug). The MUII-Plus umbrella supports trainees (investigators) including undergraduates, postgraduates, post-doctoral fellows, and emerging research leaders.

At the start of the MUII-Plus programme, we realised that a number of trainee investigators had limited knowledge of procedures governing research and how to conduct their projects correctly. To equip the investigators with the necessary skills and promote scientific quality, MUII-Plus launched routine monitoring of research sites and activities for all their investigators in April 2017. 

In this paper we present a model for internal monitoring of trainee investigators’ research projects that we have found achievable and effective in a local academic research setting. We believe this model can be adopted by other training programmes to benefit and support the progress of their investigators.

## Methods

### Site monitoring processes

Routine monitoring of research projects for all MUII-Plus investigators commenced in April 2017 to date. This involves internal monitors reviewing and evaluating investigators’ research sites and projects based on a standard checklist ( Table 1). In this study, we report findings for monitoring done between April 2017 and December 2019.

Four monitoring visits were planned for each research project; site initiation (SIV), two interim (IMV), and a closeout monitoring visit (CMV). A team of two (MA and SC) conducted the monitoring visits. MA, a registered midwife, worked as a research nurse for nine years, then trained as a clinical trial monitor in 2011 under the East African Consortium for Clinical Research (EACCR), and was certified as a Clinical Research Associate (CRA) in 2017 by African Clinical Research Organisation (ACRO); she is experienced in monitoring observational studies and clinical trials. MA was assisted by SC, a registered nurse with a 15-year experience; SC also trained as a Clinical Trial Monitor under EACCR.

**Table 1.** Items included in the MUII-Plus trainee’s monitoring checklist.

Reviewed documents
1. Institutional Review Board & Uganda National Council for Science and Technology approval/favourable opinion notification
2. List of members of Ethic Committee
3. Administrative letter from the study site (e.g. hospital; if applicable)
4. Signed approved protocol (and all amendments)
5. Stamped consent/assent forms & all translations (including translation certificate)
6. Subject recruitment material e.g. briefing/information slides, participant handouts, adverts for subject recruitment such as radio, TV & other media adverts
7. Blank copies of Case Report Forms (CRFs), source documents, lab request forms, master Serious Adverse Event form, protocol deviation form, screening log, enrolment log, reimbursement form etc.
8. Study financial agreement (put note to file if this is filed elsewhere)
9. Insurance statement for research related injury (if applicable)
10. Study staff training records e.g. protocol training, Standard Operating Procedure (SOP) training, source document training, CRF & electronic (e)CRF training records,
11. Updated signed Curriculum Vitae for each study staff
12. Certificate of qualifications
13. Updated signed Job Descriptions
14. GCP/HSP certificate
15. GCLP for lab personnel in addition to the above certificate
16. Annual Practice Licenses (APL) (where applicable)
17. Study monitoring plan
18. Site monitoring log
19. Site Initiation Visit (SIV) agenda
20. Site Initiation Visit Report
21. Interim monitoring agenda
22. Interim monitoring report
23. Close out monitoring agenda
24. Close out monitoring report
25. Delegation of Duties (DoD) Log
26. Site staff contact details list
27. Study quality management plan
28. Participant flow chart
29. Communication flow chart
30. Lab accreditation certificate if applicable
31. Laboratory analytical plan if applicable
32. Material Transfer Agreement if applicable
33. Study specific SOPs
34. MUII-Plus engagement plan
35. MUII-Plus award letter/acceptance letter
36. Meeting minutes with supervisor and study team
37. Gantt chart
Inspection of facilities
1. Adequate facilities for all study related procedures
2. Site has received all supplies required to conduct the study
3. Adequate facilities for storage of samples

For each new study, the monitors and investigator (trainee) discussed, planned, and shared a list of essential documents to be reviewed at least a week before the first monitoring referred to as the site initiation visit (SIV). Once the SIV date was confirmed, the monitors sent a monitoring agenda to the investigator before the visit.

### Site initiation visit (SIV)

The SIV was to establish research sites and facilities to ensure investigators had all the necessary approvals, qualified and skilled staff, data collection tools and documents, and laboratory materials to implement the proposed research project.

During this visit, the investigator was asked to share and explain his or her project proposal, clinical, laboratory, or pharmacy procedures as applicable, and data management plan. Similarly, the monitors informed investigators about the purpose of the monitoring, the monitors’ and investigator’s responsibilities, informed consent procedures, and good documentation practices. Additionally, the monitors critically verified the investigator’s site file (ISF) which comprised of academic documents, approved protocols, valid practicing licenses, GCP, and GCLP certificates for staff as applicable ( Table 1). In case of any queries, the investigator was given time to address them and a SIV follow-up visit was done for corrective action before the project commenced.

### Interim monitoring visit (IMV)

The IMV followed the SIV intending to review the progress of the commenced project. First, the monitor checked whether the investigator screened and enrolled participants, collected, documented, and managed data as described in the approved standard operating procedures (SOPs) and protocols. For data management, the monitor verified that the completed data collection forms or source data matched that entered in the database and backed up routinely. Second, the research site was toured to ensure adequate and proper use of research materials, procedure rooms, and storage facilities for specimens and samples, documents and drugs as applicable. The IMV was concluded with a discussion on the key issues identified and the investigator was advised on the appropriate action to address the issues, as the investigator awaited a detailed visit report.

### Study closeout monitoring visit (CMV)

The CMV was performed at the end of the research project when all study participants’ visits and follow-up were complete and all data collected as required. Here, the monitor revisited the ISF, consent forms, data collection forms, and databases, to ensure that all were complete. In addition, the monitor and investigator planned for proper storage of study documents and samples to enable easy retrieval for future use.

### Monitoring report

After each visit, a written report was shared with the investigator and his or her supervisors for review and signing. Then the monitor co-signed the final report and shared it with the investigator, MUII-Plus programme centre manager and director. In case of any critical findings that could not be resolved between the trainee and monitor, the director or centre manager would have meetings and discuss the way forward with the investigator.

### Model used to review the monitoring reports

To assess compliance of MUII-Plus investigators to Good Clinical Practice, the monitoring team considered six elements: (1) regulatory documents, (2) informed consent process and documentation, (3) protocol adherence and Source data verification (SDV), (4) study-related training, (5) working practices and (6) tour of project site facilities ( Table 2).

**Table 2.** General Monitoring Activities conducted for all the four visits.

Item	Essential document for review	Observations
1. Regulatory documents	Approved protocols	Availabilities of study related documents
	Informed consent / assent forms and wavier of consent if applicable	
	Approval letters from Research Ethics Committees (REC)	
	Case Report Forms	
	Annual Practice Licenses (APL)	
	Curriculum Vitae and academic documents	
2. Informed consent documentation and participant status	All the screened and enrolled participants have signed and dated copy of current approved ICFs prior to any study-related procedures being conducted	Observe the process of obtaining informed consent forms
	Investigators maintain logs of screened and enrolled participants within the study	
	Storage consent forms available for all samples stored in the freezers or waiver of consent if applicable	
	Amount of reimbursement approved in consent forms given to participants and documented	
3. Protocol adherence and Source Data Verification (SDV)	Source documents and other study records are accurate, complete, and up-to-date, and check the accuracy and completeness of the case report form entries	Observe protocol deviation
4. Study related training	Protocol and SOP training records, source documents/case report form training	Ability to perform as trained
	Updated GCP/GCLP certificates	Training certificates
	Protection of Human Research Participants (PHRP) Certificates	
5. Working practices	Availabilities of SOPs and delegation and responsibility log	SOPs at work station
	Minutes of meetings with Supervisors and study team if applicable	Frequency of meetings
6. Tour of project site facilities	Clinic room, Laboratory process area and data management area and pharmacy facility	Adequate facilities for study related procedures and storage of records and study drugs
	Study reagents and materials	Site has received all supplies required to conduct the study
	Storage facilities for specimens collected and study drug if applicable	Adequate facilities for storage of samples and study drug if applicable

Regulatory documents are guidelines that the monitor uses to keep the investigator within the legal and ethical boundaries during their research projects, and assess the research conduct and quality of data generated. These included approved protocols and consent documents, data collection forms, curriculum vitae, academic documents and others, as described in ( Table 2).

Obtaining informed consent from participants is very important in the ethical process of human research. This process requires that the investigator respects and protects the rights of the participants by thoroughly explaining the research objectives and expected requirements from participants before obtaining their consent. All participants sign and date on the consent form as proof of their consent to enroll in the research. After this, a copy of the form is shared with the participant. Throughout the project, the investigator and participant maintain information exchange, and the participants reserve the right to withdraw their consent.

Protocol adherence and source data verification requires that the investigator adheres to the approved protocols to ensure data generated and captured is accurate and complete.

An investigator and their staff must undergo thorough training on different aspects of the proposed research project, including GCP/GCLP guidelines and SOPs, so that they are competent in their work. Often, members are awarded certificates on completion of the trainings which they put on file.

Evaluation of working practices involves assessment of teamwork and coordination between research investigators and staff for effective communication and implementation of the research project. For example, tracking the number of times trainee investigators meet their supervisors and checking whether meeting minutes are on file. All these aspects were evaluated based on whether documentation was present at each site visit.

### Data extraction and analysis

We extracted data on components monitored from the approved and signed off monitoring reports from each visit. The data was entered into an excel spreadsheet with each variable representing an item in Table 2. For each project, a score of one (1) was assigned to each item if its documentation/facility was present and zero (0) otherwise. An average score was obtained and converted into a percentage compliance for every visit. We used Stata version 15.0 (StataCorp, College Station, USA) for analysis.

### Ethics and consent

This report describes the findings of an internal evaluation undertaken to support learning, following the implementation of internal monitoring to enhance the quality of work undertaken by research trainees. The work was reviewed by the Research Ethics Committee of the Uganda Virus Research and a determination of “non-research”, waiving the requirement for ethical review and approval, was made. All the investigators gave written permission for the reports on their work to be used for this evaluation and publication.

## Results

**Figure 1.**
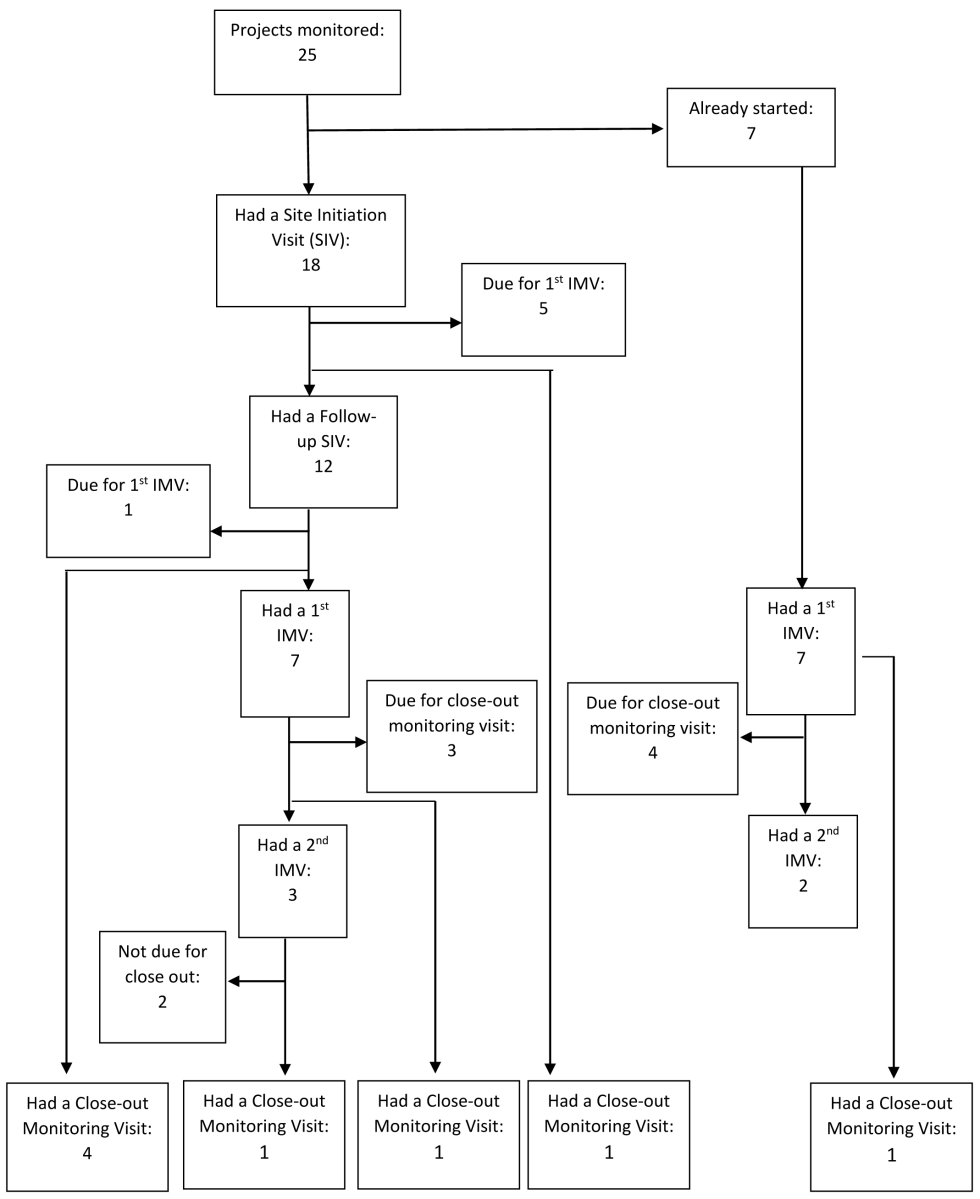
Projects monitored at each monitoring visit.

We reviewed documents and reports for masters, PhD, and post-doctoral fellows’ projects running between April 2017 and December 2019. During this period, there were 26 research projects, and we monitored 25 (96.2%) of these. Of the monitored studies, 18 underwent a site initiation visit (SIV), 12 underwent SIV follow-up, 14 had the first interim monitoring visit (IMV), 5 had a second IMV, and 8 had a closeout monitoring visit (CMV) by the time of analysis. Some studies did not have all monitoring visits because they started earlier than the monitoring programme ( Figure 1).

### Regulatory documents

During the SIV, 43% of the projects were compliant based on regulatory documents. The compliance was lower than expected because investigators had not obtained project or protocol approvals from the different Research Ethics Committees at the time of analysis. One study lacked regulatory documents on file, and it was hard to determine whether it had valid approvals and was compliant in other administrative aspects. At this time, none of the projects that planned to ship biological samples had obtained the material transfer agreements (MTA) required. We also observed poor documentation practice: for instance, many investigator files did not have a table of contents, and it was difficult for the monitor to identify and access filed records quickly.

However, the compliance improved to 77% at the time of the SIV follow-up visit. There was an improvement of 92% and 100% during the second interim and final closeout visits, respectively ( Table 3 ^8^).

**Table 3.** Performance of investigators at each monitoring visit.

	Mean percent (%) compliance of investigators				
	Site Initiation Visit (SIV)	SIV Follow-up Visit	1 ^st^ Interim Monitoring Visits (IMV1)	2 ^nd^ Interim Monitoring Visit (IMV2)	Close-out Monitoring Visit (CMV)
Number of investigators assessed	n=18	n=12	n=14	n=05	n=08
1. Regulatory documents	43	77	70	92	100
2. Informed consent documentation and participant status	44	75	93	100	100
3. Study related training	31	75	54	90	100
4. Working practices	28	50	50	100	100
5. Tour of project site facilities	81	92	90	100	100
**Average compliance across** **all domains**	**45**	**73**	**71**	**96**	**100**

### Informed consent process and documentation

There was 44% compliance with the informed consent process and documentation at SIV. Sometimes essential documents such as the informed consent forms were still being developed or under consideration by the ethics committees. Compliance levels increased during the following monitoring visits, 75% at SIV follow-up and 93% at first IMV. At these visits a few projects had incomplete or missing consent forms and in some cases research staff had signed as witness for participants (contrary to good practice). 

By the second IMV and CMV, all projects (100%) were compliant with complete consent forms and documentation ( Table 3).

### Protocol adherence and source data verification (SDV)

Overall, the majority of the investigators adhered to their research protocols and standard operating procedures, and data collected was accurate and complete.

### Study-related training

Only 31% of investigators had evidence of study-related trainings at the SIV because most of them had not received training on GCP/GCLP guidelines and SOPs. On subsequent monitoring, 75% and 54% of investigators and their staff had been trained and certified at the SIV follow-up and first IMV, respectively. Towards the last monitoring visits, we achieved 100% compliance ( Table 3).

### Working practices

Under working practices, compliance was 28% at the SIV and improved to 50% at both the SIV follow-up and first IMV. Some investigators had held and documented study-related meetings regularly. A few had no meetings at all at SIV. During the second IMV and CMV, full compliance of (100%) were recorded ( Table 3).

### Tour of project site facilities

While carrying out the tour of project site facilities, the monitors focused on the clinic room, laboratory process area, data management area, study reagents and materials availability, storage facilities for specimens collected, and study drugs.

The majority of the research sites had facilities that were adequate to conduct the studies. The facilities complied with the minimum standards described in Table 2 at 81% during the site initiation visit and above 90% for the subsequent monitoring visits ( Table 3). However, we noted congestion at participant recruitment stations.

### Challenges encountered by monitors

The monitors encountered logistical delays from investigators in confirming appointments for monitoring, reviewing and giving feedback on monitoring reports, and addressing monitoring issues raised.

## Discussion

We have presented an internal evaluation of the MUII-Plus research monitoring programme. Our findings show that many trainee investigators, and their research teams, needed training in good clinical research practice – to an extent that we had not recognised at the start of our programme. Through internal monitoring, we recognised the needs of investigators and trained them, which improved their compliance with the research guidelines. We believe that these findings highlight a critical training need, and we present a monitoring model that could contribute to advancing research excellence across Africa.

The reviewed reports emphasized the need for investigators to pay close attention to the regulatory requirements, especially ethical approvals for their research projects, and the monitors to carry out a pre-site assessment visit to minimize non-compliance observed during the site initiation visits. Our findings reflect experience across the continent: in one example, only 9.8% of student dissertations on HIV across universities in Cameroon ^9^ documented ethical approvals. There is a need to address the lack of knowledge in both students and their mentors about principles guiding human research, and requirements for documentation of approval processes.

Informed consent is an aspect of ethical human research that needs keen attention. A study done in Uganda between 2007 and 2010 showed that 36% of research sites violated the informed consent process ^7^. We found that, at first, the informed consent process was not adequately practiced by some investigator trainees in the MUII-plus programme: some projects had incomplete consent forms, project staff signed as witnesses for participants, signed copies were not given to participants, and occasionally forms were missing. However, our continuous monitoring showed improved compliance up to 100% at the second IMV and CMV. The marked improvement observed in our study implies that consent processes during investigators’ projects can be improved by prior training and sensitization of investigators and their study teams and frequent monitoring.

During the site initiation visit, compliance in terms of providing research teams with study-related training and adopting good working practices, such as regular team meetings, was low, 31% and 28%, respectively. Here, GCP/GCLP certificates and protocol training logs for team members, and minutes for supervision or team meetings, were lacking. This low compliance was because the trainee investigators lacked knowledge on the kind of team trainings they were supposed to undertake. This prompted the MUII-Plus programme to fully fund face-to-face GCP/GCLP training for all investigators and their teams in 2017 and 2018. Following the first training, all investigators undertake a refresher online GCP/GCLP training, such as the course hosted by the Global Health Training Centre (GHTC) ^10^, every two years. Investigators must learn the importance of providing protocol and SOP training for their team before research work commences, and be involved in team-building activities and regular team meetings to maintain effective communication and implementation during the research activities.

Site facilities for our trainees were found to be relatively adequate concerning clinic rooms, laboratory process areas and data management areas, study reagents and materials availability, and storage facilities for specimens collected and study drugs. Not surprisingly, given the setting of busy African hospitals and clinics, a good number of investigators faced the challenge of congestion at recruitment locations, due to limited space.

Reviews of protocol adherence and source data verification were reassuring: the data collected was generally accurate, and complete.

During the CMV, we observed that among some studies that collected samples the investigator lacked a proper plan for longer-term sample and document storage. This is a significant challenge that needs to be faced by African institutions for their trainees and research teams.

Undertaking research as a post-graduate student or post-doctoral researcher is a challenging process with many competing demands on trainees’ time. This must have contributed to the challenges faced by monitors in scheduling their work. Institutional buy-in and a research culture that supports quality and rigour in compliance with human subjects research guidelines is needed to support an effective internal monitoring programme. 

## Recommendations

Through the MUII-Plus programme monitoring, we have learnt the importance of inducting and training investigators and their teams on GCP/GCLP guidelines, the informed consent process, and protocols before the research activities begin. We urge that Universities and research institutes across Uganda and Africa prioritise these trainings to staff and students before allowing them to embark on any human research project. Institutional research ethics committees should enforce GCP/GCLP training as a requirement for project approval.

## Conclusions

The MUII-Plus programme’s monitoring model has improved the confidence and quality of the research output of the investigators tremendously. Routine site monitoring is a successful tool to identify gaps in research training and implementation, and improve the quality of research. Research site monitoring should be introduced and implemented across research institutions in Africa.

## Data availability

### Underlying data

LSHTM Data Compass: Internal monitoring within MUII-plus for research capacity development. https://doi.org/10.17037/DATA.00001938 ^8^

This project contains the following underlying data:

- Project_monitoring_data_XLSX.xlsx (A dataset containing data provided by 25 projects for an internal monitoring evaluation of the MUII-plus research programme)

Data are available under the terms of the Creative Commons Attribution 3.0 Unported license (CC-BY 3.0).

## References

[1] Council for International Organizations of Medical Sciences: International Ethical Guidelines for Biomedical Research Involving Human Subjects.2002. [Accessed 4th May 2020]. Reference Source 14983848

[2] Uganda National Council for Science and Technology: National Guidelines for Research involving Humans as Research Participants.2014. [Accessed 4th May 2020]. Reference Source

[3] WHO: Guidelines for good clinical practice (GCP) for trials on pharmaceutical products.1995. [Accessed 4th May 2020]. Reference Source 10.3109/07853899409147334 8024733

[4] WHO: Operational guidelines for ethics committees that review biomedical research.2000. [Accessed 4th May 2020]. Reference Source

[5] World Medical Association: World Medical Association Declaration of Helsinki: ethical principles for medical research involving human subjects. *JAMA.* 2013;310(20):2191–2194. 10.1001/jama.2013.281053 24141714

[6] NIH: DAIDS Guidelines for Good Clinical Laboratory Practice Standards.2019. [Accessed 16th July 2020]. Reference Source

[7] Ochieng J Ecuru J Nakwagala F: Research site monitoring for compliance with ethics regulatory standards: review of experience from Uganda. *BMC Med Ethics.* 2013;14:23. 10.1186/1472-6939-14-23 23738971 PMC3683324

[8] Akello M Coutinho S Mboowa MGN: Internal monitoring within MUII-plus for research capacity development. [Data Collection]. London School of Hygiene & Tropical Medicine, London, United Kingdom.2020. 10.17037/DATA.00001938

[9] Munung NS Tangwa GB Che CP: Are students kidding with health research ethics? The case of HIV/AIDS research in Cameroon. *BMC Med Ethics.* 2012;13:12. 10.1186/1472-6939-13-12 22686445 PMC3470971

[10] Global Health Network: ICH Good Clinical Practice E6 (R2). [Accessed 16th July 2020]. Reference Source

