# Supplementary material for: Continuous research monitoring improves the quality of research conduct and compliance among research trainees: internal evaluation of a monitoring programme
Source: AAS Open Res. 2020 Nov 25;3:57. [Version 1] doi: 10.12688/aasopenres.13117.1 (PMC8170533; doi:10.12688/aasopenres.13117.1)
Supplement: Peer review history

# Peer review: Reviewer response for version 1

**DOI:** 10.21956/aasopenres.14217.r28611

**Published:** 2021-06-01

**Authors:** Victoria Yorke-Edwards

**Affiliations:** 1 MRC Clinical Trials Unit, Institute of Clinical Trials and Methodology, University College London, London, UK

**Main comments:**

 This paper has the potential to be a valuable contribution to the discussion of how best to support the training of new clinical trial investigators. It details a programme of monitoring of the studies of trainee Investigators by the Makerere University/Uganda Virus Research Institute Centre of Excellence in Infection and Immunity Research and Training (MUII-Plus), giving details of monitoring findings across the programme and proposing it as a potential, practical, model for similar institutes. The results and discussion highlight several findings that should be of concern to all those involved in Investigator training, and highlight the utility of monitoring not only to the research studies themselves, but also to wider investigator training.

 However, I think that it would benefit from some careful clarification of its background and aims sections (and abstract) to allow potential readers to appreciate the subject matter that it deals with. At present the title and above named sections do not accurately convey the nature of the paper. As it is, the reader only really realises the full scope of the paper part way through the Methods or even into the Results section. This is a shame, as the results section provides strong evidence of the need to provide experienced monitors, independent of the research project, to inspect new Investigator’s first research studies, and provide guidance and training at the appropriate point to ensure regulatory compliance, patient safety and wellbeing, and study integrity. The authors also point to some extremely important findings regarding the training of new Investigators (especially in GCP/ GCLP) before they even embark on a research study with human subjects. Slight changes to the abstract and background would much better set the scene for these important observations.

 I also have some concerns over the simplicity of the analysis, which is very simple, and perhaps overly basic. Scoring was binary, with a point awarded for each item if a document or facility was present (zero if not). The presence of a point therefore does not necessarily indicate that the quality of that document or facility was adequate, or at least, the reader is not told that the scoring took into account quality. This leaves the question of whether, say, a point would have been awarded if the delegation of duties log had been present but not up-to-date, or a monitoring plan in place but no evidence of it being followed. I would therefore like to see an improved manuscript that explains in more detail the scoring, and includes a good discussion of the limitations of this approach.

 Overall, the paper proposes a useful approach to supporting new Investigators with their first clinical studies, and highlights some important gaps in Investigator training. However, several key issues with the current manuscript need to be resolved.

**Other issues:**

1. The terminology used can be a little confusing especially at the beginning of the paper when the reader is trying to work out what it is about. This is possibly in part because of the difficulty of finding terminology to fit in a field in which “monitoring” already has such heavily used but varied meanings. Here, monitoring is carried out by those external to the actual study (I’ll call them programme monitors hereafter) to assist in the training of new Investigators, and to ensure that their research studies are being carried out appropriately. The use of “internal monitoring” is therefore somewhat confusing. I would suggest that either the terminology is changed to something more appropriate, or a clear definition is set out early in the paper e.g. “ *To equip the investigators with the necessary skills and promote scientific quality, MUII-Plus launched routine monitoring of all investigator’s research sites and activities in April 2017, hereafter termed “internal monitoring*”. (Note the slight rewording for clarity!).
2. On a similar note, the use of the phrase “continuous monitoring” could lead to confusion with real-time monitoring methods such as central monitoring of the trial database, so I would suggest removing the word “continuous” or changing it to “periodic”.
3. Also, the first few paragraphs seem to confuse/conflate checks by Ethics Committees with site monitoring. I would like to see this rewritten to ensure that what is being stated is clear and appropriately referenced. I was surprised to see “and monitored as per international human research regulatory guidelines” being referenced with the *Operational guidelines for ethics committees that review biomedical research*, which discusses ethical review. I was also surprised not to see ICH GCP E6 (R2) only being referenced by way of the course suggestion at the end of the paper (reference 10): it has a lot to say on monitoring!
4. I assumed from the mention of GCP that the research studies being monitored were clinical trials, but this is not explicitly stated. It would be useful to state in the Background which types of studies the Investigators were running e.g. clinical trials, single-site, intervention type.
5. The background section states that the paper will present a model for “internal monitoring” but I think it should also explicitly state that the paper will detail the findings from testing this model out in the MUII-Plus Programme. Similarly, the Methods section should discuss in more detail how the model was evaluated across the programme: i.e. this section shouldn’t just describe the model itself but how the results will be assessed across the whole programme to determine the value of this kind of monitoring to training programmes such as MUII-Plus. (See also my comments on the point system that was used, above).
6. Table 1 indicates that the programme monitors’ checklist included checks of the monitoring documents for each study but there is no mention of checks of monitoring in Table 2 nor in the Results section. It would have been helpful to see some discussion of the monitoring strategies being employed for the studies by the Investigators themselves, who was carrying them out and what they broadly entailed, and any findings from the programme monitors. As the Background states, monitoring is an essential part of human studies so the emission is a big one in a paper that is basically about monitoring.
7. The percentages quoted in the Results section can be a little misleading as sometimes they are obtained from few data points. It would be helpful to give a sense of the number of sites involved for each percentage e.g. “There was 44% compliance with the informed consent process and documentation at SIV” could become “There was 44% (8/18 studies) compliance with the informed consent process and documentation at SIV”. For that matter, it is not always clear what is being discussed. For example, in the Regulatory Documents sub-section there is the statement: “During the SIV, 43% of the projects were compliant based on regulatory documents”, but if that meant 8/18 studies that had the SIV, how is the percentage 43% and not 44%? It leaves the reader wondering whether they know all they need to know about how compliance was assessed. Describing nominators and denominators may help.
8. I would have liked to see a limitations section in the discussion, and details of any future plans: is MUII-Plus continuing the monitoring programme and will they continue to evaluate it? If so, what might they do differently?
9. I think Figure 1 is useful but would suggest that any good figure should include all the information needed to interpret it. I therefore suggest that any acronyms are explained either in the Figure itself or the Figure label.
10. I am pleased to see that the data has been shared online, and that a user guide has been supplied. I do think that it would be helpful to explain a few more of the variables in the user guide however, e.g. those that include acronyms such as APL.

Is the work clearly and accurately presented and does it cite the current literature?

Partly

If applicable, is the statistical analysis and its interpretation appropriate?

Partly

Are all the source data underlying the results available to ensure full reproducibility?

Yes

Is the study design appropriate and is the work technically sound?

Yes

Are the conclusions drawn adequately supported by the results?

Partly

Are sufficient details of methods and analysis provided to allow replication by others?

Partly

Reviewer Expertise:

Clinical Trial Conduct Methodology; Monitoring; Data Sharing

I confirm that I have read this submission and believe that I have an appropriate level of expertise to confirm that it is of an acceptable scientific standard, however I have significant reservations, as outlined above.

# Peer review: Reviewer response for version 1

**DOI:** 10.21956/aasopenres.14217.r28607

**Published:** 2021-06-01

**Authors:** Carolynn Thomas Jones

**Affiliations:** 1 Center for Clinical and Translational Science, College of Nursing, The Ohio State University, Columbus, OH, USA

I agree with prior reviewers that the terminology of Research site monitoring (and its definition) implies that it is the supervisory work of investigators; however this activity seems to be the institution of an institution's internal quality monitoring focusing on trainee research projects at different stages. This is a great workforce development and research compliance activity, it would be highly instructional and important to advancing trainee research careers.

 Also, this is a method of providing capacity building to your research enterprise and the establishment of institution-wide institutional quality compliance monitoring. Were there other monitoring groups conducting monitoring (e.g., industry sponsors, government sponsors, etc.) or was this the only monitoring taking place at your institution?

Is the work clearly and accurately presented and does it cite the current literature?

Yes

If applicable, is the statistical analysis and its interpretation appropriate?

Yes

Are all the source data underlying the results available to ensure full reproducibility?

Yes

Is the study design appropriate and is the work technically sound?

Yes

Are the conclusions drawn adequately supported by the results?

Yes

Are sufficient details of methods and analysis provided to allow replication by others?

Yes

Reviewer Expertise:

My areas of research is in clinical research core competencies, workforce development, clinical research training and education, GCPs

I confirm that I have read this submission and believe that I have an appropriate level of expertise to confirm that it is of an acceptable scientific standard.

## References

[1] : Research monitoring by US medical institutions to protect human subjects: compliance or quality improvement?. *J Med Ethics*.2013;39(4) : 10.1136/medethics-2011-100434 236-41 10.1136/medethics-2011-100434 22902386

[2] : Tracking the career development of scientists in low- and middle-income countries trained through TDR's research capacity strengthening programmes: Learning from monitoring and impact evaluation. *PLoS Negl Trop Dis*.2017;11(12) : 10.1371/journal.pntd.0006112 e0006112 10.1371/journal.pntd.0006112 29216192PMC5736233

# Peer review: Reviewer response for version 1

**DOI:** 10.21956/aasopenres.14217.r28604

**Published:** 2021-05-27

**Authors:** Celeste Cagnazzo

**Affiliations:** 1 Department of Public Health and Pediatrics, Pediatric Onco-Hematology, Stem Cell Transplantation and Cellular Therapy Division, Regina Margherita Children's Hospital, University of Turin, Turin, Italy

The authors present a very interesting project, aimed at increasing the quality in the execution of clinical trials. However, I have some major considerations:

- cit n 3 refers to GCP R1 but R2 is is the current version.
- What types of studies was monitoring applied to? Interventional, observation, industry sponsored, academics?
- In case of application to industry sponsored studies, were there any differences to the monitoring normally performed by the promoter?
- Table 2: the observations are too general and not adequate to describe the real result of the monitoring. What was observed? What kind and degree of deviations?
- Figure 1 is difficult to read, it is recommended to modify it.
- To consider the preparation of the PIs adequate, was only the possession of a GCP training taken into account? Perhaps it is not enough, a continuous training program should be considered.
- Challenge encountered by monitor: how often? Detail this.
- It is not clear what types of indicators and criteria were considered. Furthermore, the description of any hypothetical corrective and preventive actions is missing. With what purpose was the project started? Just observation?

Is the work clearly and accurately presented and does it cite the current literature?

Partly

If applicable, is the statistical analysis and its interpretation appropriate?

Not applicable

Are all the source data underlying the results available to ensure full reproducibility?

Yes

Is the study design appropriate and is the work technically sound?

Partly

Are the conclusions drawn adequately supported by the results?

Partly

Are sufficient details of methods and analysis provided to allow replication by others?

Partly

Reviewer Expertise:

Clinical research methodology and management; clinical research training programs

I confirm that I have read this submission and believe that I have an appropriate level of expertise to confirm that it is of an acceptable scientific standard, however I have significant reservations, as outlined above.

# Peer review: Reviewer response for version 1

**DOI:** 10.21956/aasopenres.14217.r28232

**Published:** 2021-02-25

**Authors:** Mainga Hamaluba, Marianne Munene, Esther Kivaya

**Affiliations:** 1 KEMRI-Wellcome Trust Research Programme, Kilifi, Kenya

The Makerere University/Uganda Virus Research Institute Centre of Excellence in Infection and Immunity Research and Training (MUII-Plus) introduced internal monitoring between April 2017 and December 2019 to promote scientific quality specifically for research projects led by trainees and early to mid-career researchers. This involved up to 4 pre-determined monitoring visits across 25 out of 26 projects during the study period. They report an improvement in the quality of the data and GCP compliance and now propose a model for other research institutes to potentially adopt/adapt in order to promote quality research. Overall, the group share data on a practical implementable approach to improving compliance with GCP in clinical research for trainee/early researchers in LMIC settings. They audit this approach over 2 years and report their findings. The authors may want to consider the following items:

 There isn’t a clear description of the differences between research site monitoring (RSM) and internal monitoring and these seem to be used interchangeably. Further definition of both would be useful including who conducts RSM vs internal monitoring and the different aims of the 2 processes. The abstract refers to all investigators having good research ethics and practice but doesn’t define how this was measured. The term continuous research monitoring isn’t clearly defined and may be confused with continuous monitoring processes such as central monitoring suggesting real-time dynamic monitoring processes. We would suggest the authors simply use the regular term, research monitoring, rather than continuous research monitoring for clarity. Details on the nature of each study (clinical trial, observational study, length of the study, sample size, IMP or registered products etc.) would be useful to contextualise the applicability of this approach to different study designs of varying complexity. The utility of this approach may vary depending on these factors. It may be worth commenting on other factors that may have affected the monitoring process; any changes in the regulatory landscape in the period that the studies were evaluated, e.g. new submission requirements, how many protocols had amendments to their procedures, staff turnover etc.

 A clear description of what scoring criteria were used to quantify rates of compliance for each monitored item isn’t available. The evaluation and comments on the effect of monitoring should be interpreted cautiously as the required SIV, 2 IMVs and CMV were not conducted across all the studies with a relatively small sample size. Figure 1 requires further clarification. The outcome of those due a visit (IMV or close out) isn’t clear, did these visits occur? For those that didn’t have sequential visits i.e. had 1 rather than 2 IMVs, it is not clear why this was the case. Table 3 could be made clearer. It is unclear if the number of investigators assessed reflect the number of studies monitored, this should be clarified.

 It’s unclear if there was a monitoring plan for each study independent of the 4 visits. The authors may want to consider/discuss how this monitoring approach compares/interacts with other monitoring approaches particularly in LMIC settings (e.g. risk-based approach, central, remote monitoring etc.) and benefits and disadvantage of this approach versus others.

 It would have been useful if there was a key or breakdown of the scoring for compliance as per the six domains (table 2), noting that in table 3 there were on results on 5 domains.

 Do the authors have observational data on studies that had not been monitored using this approach throughout their lifecycle according to the criteria in table 1 and 2 for comparison? This would strengthen any findings.

 Overall, proposes a pragmatic approach to trainees/early researchers improving compliance to GCP and the quality of their research outputs using the proposed monitoring tools but there are several minor clarifications that could benefit from further details as suggested above.

Is the work clearly and accurately presented and does it cite the current literature?

Yes

If applicable, is the statistical analysis and its interpretation appropriate?

Yes

Are all the source data underlying the results available to ensure full reproducibility?

Yes

Is the study design appropriate and is the work technically sound?

Yes

Are the conclusions drawn adequately supported by the results?

Partly

Are sufficient details of methods and analysis provided to allow replication by others?

Partly

Reviewer Expertise:

Clinical research, Clinical trials, Monitoring

We confirm that we have read this submission and believe that we have an appropriate level of expertise to confirm that it is of an acceptable scientific standard, however we have significant reservations, as outlined above.
